# Possible Anti-inflammatory Effects of Mesenchymal Stem Cells Transplantation via Changes in CXCL8 Levels in Patients with Refractory Rheumatoid Arthritis

**DOI:** 10.22088/IJMCM.BUMS.8.3.191

**Published:** 2019

**Authors:** Arezoo Gowhari Shabgah, Zhaleh Shariati-Sarabi, Jalil Tavakkol-Afshari, Mohsen Ghoryani, Mojgan Mohammadi

**Affiliations:** 1 *Immunology Research Center, Mashhad University of Medical Sciences, Mashhad, Iran.*; 2 *Immunology Department, Mashhad University of Medical Sciences, Mashhad, Iran.*; 3 *Rheumatic Diseases Research Center, Mashhad University of Medical Sciences, Mashhad, Iran.*; 4 *Department of Laboratory Sciences, School of Paramedical Sciences, TorbatHeydariyeh University of Medical Sciences, Torbat Heydariyeh, Iran.*; 5 *Research Center of Advanced Technologies in Medicine, Torbat Heydariyeh University of Medical Sciences, Torbat Heydariyeh, Iran.*; 6 *Allergy Research Center, Mashhad University of Medical Sciences, Mashhad, Iran.*

**Keywords:** Rheumatoid arthritis, mesenchymal stem cells transplantation, CXCL8, CXCL12, CXCL13

## Abstract

The synovial- lining cells have been involved with rheumatoid arthritis (RA) through the secretion of various cytokines and chemokines. Increased levels of these cytokines and chemokines are seen first in the synovial and subsequently in the bloodstream of RA patients. The synovial and circulating levels of CXCL8, CXCL12, and CXCL13 are higher in the RA patients than in the healthy subjects, causing migration of immune cells to the joints, which is associated with increased joint destruction. We aimed to evaluate the effects of autologous mesenchymal stem cells intravenous administration on plasma levels of CXCL8, CXCL12 and CXCL13 at 1, 6, and 12 month follow-up periods in refractory RA patients. 13 patients with refractory RA received autologous mesenchymal stem cells (MSCs). The ELISA technique was used to evaluate the plasma level of these chemokines. CXCL8 levels were significantly decreased at month 6 after MSCs transplantation in comparison with pre-injection level, and the concentration of this chemokine was significantly increased at month 12 in comparison with the month 6 after injection (P <0.05). The levels of CXCL12 and CXCL13 were insignificantly decreased at months 1 and 6 after the MSCs transplantation. The interaction of MSCs after migration to the inflamed joints with CXCL8-producing cells could be one but not the only possible mechanism that reduces its production in the joints and subsequently in the plasma of RA patients. CXCL8 reduction as a consequence of MSCs application returned to pre-injection levels after 12 months. Therefore, increasing the dose of MSCs and replication of injections may maintain the potential anti-inflammatory effects of MSCs on the production of CXCL8 as an inflammatory mediator in patients with refractory RA.

Rheumatoid arthritis (RA) is a chronic systemic disease characterized by the simultaneous inflammation of the synovium of multiple joints, leading to cartilage and joint damage, which affects 1% of people around the world, decreases the life expectancy, and results in chronic disability. The inflmation and synovial hypertrophy are the common processes in the RA. The etiology of the disease is unknown, but immunological abnormalities are involved in the pathogenesis of the disease. Accumulation of B/T lymphocytes, neutrophils, macrophages and monocytes is found in the synovial tissues. The lymphocytes and cytokines are effective in localizing and activating immune cells, endothelial cells and fibroblasts, as well as in enhancing inflammatory response (1).

Cell therapy has been shown to be a remarkable potential for the treatment of many diseases in recent years (2-6). Among these, there is a great hope for the use of mesenchymal stem cells (MSCs). Some of the reasons for the widespread use of these cells in cell therapy are their ease of extraction, ability to differentiate into different types of cells, paracrine effect, immune-modulating properties, and migration behavior (3, 5).

Establishment is a rapid process lasting from a few hours to a maximum of one to two days, which makes temporary retention of cells in a site and does not need cell division as opposed to cell transplantation (7-9). The stromal cell- derived factor 1 (SDF1), also known as C-X-C motif chemokine 12 (CXCL12) and CXCL13 (also called BCA-1 or BLC) are B lymphocyte-specific chemokines, which act through their own specific receptors on the surface of B lymphocytes (CXCR4 and CXCR5, respectively), and cause the tissue gathering of B lymphocytes and the establishment of synovial germinal centers in RA(10). In addition, CXCL12 enhances the migration of CD27^+^ memory B lymphocytes (11). CXCL12 is expressed in bone marrow, skin, heart, liver, lung and brain endothelium. The main cause of this tissue distribution is thought to be the role of this chemokine in the recruitment of mature and immature leukocytes to these tissues (12, 13). CXCL13 and its receptor exert important roles in the B cells organization in lymphoid tissue follicles, and CXCL13 is highly expressed in the spleen, lymph nodes, gut and liver. It was shown that the synovial and plasma levels of CXCL12 and CXCL13 were significantly higher in RA patients than in healthy controls (14).

CXCL8 is particularly involved in the chemotaxis of immune cells, especially neutrophils and lymphocytes, along with stimulation of angiogenesis (15). CXCL8 as an inflammatory mediator, is expressed in cells like fibroblasts and monocytes, and acts as a chemoattractant for neutrophils, causing the gathering of neutrophils at inflammation sites such as the inflamed joints of RA patients. The synovial and circulating levels of these chemokines were significantly increased in RA patients (16). Secretion of chemokines and cytokines by resident cells in RA synovial recruits more immune cells to the site of inflammation, and raises the presence of these chemokines in the joint and then in the bloodstream. The aim of this study was to investigate the influence of intravenous injection of autologous MSCs on the levels of CXCL8, CXCL12, CXCL13 and their effects on inflammation following MSCs transplantation in patients with refractory RA.

## Materials and methods

**Study population**

According to the American college of rheumatology (ACR) and the European league against rheumatism (EULAR) 2010 RA classification criteria (17), 15 patients with refractory RA were selected from the rheumatology ward of Imam Reza Hospital affiliated to Mashhad University of Medical Sciences, Iran. All conventional drugs were ineffective on the nominated refractory RA patients in this study, and their disease was flaring. After receiving written informed consent, all patients joined the study and received all their conventional medications during the follow up period without any change in the type and dose of their medication. All participants took sulfasalazine (<1-2 g/day), prednisolone (<10-15 mg/day), and/or hydroxychloroquine (<400 mg/ day), and/or methotrexate (7.5-25 mg/week). Approval of the Ethics committee of Mashhad University of Medical Sciences for this project was IR.MUMS.REC.1395.548. Our study was designed according to the use of the archived plasma samples from the previously approved clinical trial with registration code in Iranian registry of clinical trials (IRCT) and ClinicalTrials.gov (18). Clinical and para-clinical examinations including visual analog scale (VAS) score, disease activity score-28 for RA with erythrocyte sedimentation rate (DAS28ESR), erythrocyte sedimentation rate (ESR), and C-reactive protein (CRP) were performed based on patients visit by a rheumatologist at months 1, 6, and 12 after the injection of MSCs.VAS is a pain rating scale that starts from no pain to worst, and patients had a self-assessment to indicate how they were feeling about their pain score. DAS28ESR, is a combined index of VAS and ESR to measure the disease activity which shows the tenderness and swollen of 28 joints. ESR is a laboratory test to measure the speed of red blood cells’ precipitation in the bottom of the test tube and faster rate than normal shows an inflammation status in the human body. Moreover, ESR indicates the disease activity in patients with RA. On the other hand, CRP as an important acute phase protein is a laboratory indicator which increases up to 3000 times more than normal levels in inflammation, necrosis, cancers, some of the autoimmune diseases and various infections. Measurement of CRP levels helps rheumatologists to recognize the inflammation in patients with RA (19-21).

**MSCs preparation and blood collection**

To extract the autologous MSCs, bone marrow aspiration of the patients was carried out. The processes of isolation, cultivation and injection of MSCs have been described previously (18). Briefly, after receiving 50 ml of bone marrow from patients' iliac bones, the mononuclear cells were isolated by Ficoll density gradient centrifugation (Cedarlane, Canada), and cultured in 75 cm^2^ flask (SPL, South Korea) with alpha minimum essential medium (alpha-MEM, Caisson, USA) containing 10% fetal bovine serum (FBS, Gibco, USA) and 1% penicillin-streptomycin (Caisson, USA) in a humidified incubator at 37 °C under 5% CO_2_. The supernatant was renewed every three days and all cells were harvested during 3 to 4 weeks, after reaching 80% confluence. All patients were injected once intravenously with 1 × 10^6^ MSCs per kg of body weight.

**Enzyme-linked immunosorbent assay**

An enzyme-linked immunosorbent assay (ELISA) was employed to measure the plasma levels of CXCL8, CXCL12 and CXCL13 chemokines. Before injection and at follow-ups of 1, 6 and 12 months after injection, 10 ml of blood samples were collected in EDTA anticoagulant-coated tubes for plasma collection. ELISA kits were purchased from Cloud-Clone Corp.,USA to measure plasma levels of CXCL8 (SEA080Hu), CXCL12 (SEB122Hu) and CXCL13 (SEB601Hu) according to manufacturer's instructions.

**Statistical analysis**

Data analysis was performed using generalized estimating equations (GEE) by IBM SPSS Statistics 21 (IBM Corp, USA). The results are expressed as mean ± standard error of mean (mean ± SEM), and P value less than 0.05 was considered to indicate a statistically significant difference. The GraphPad Prism software Version 8 was used for generating graphs.

## Results

**Fig. 1:**
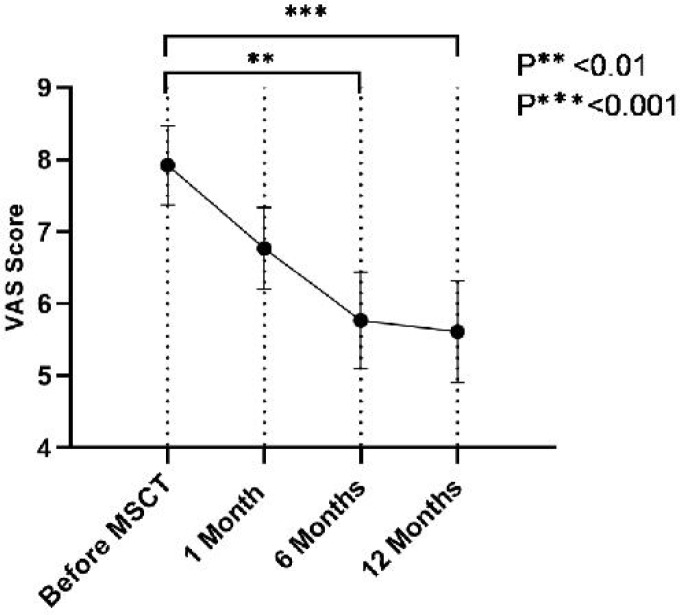
**VAS score before and after mesenchymal stem cells transplantation in RA patients.** Data are presented as mean ± SEM

The study consisted of 15 patients with RA, but only 13 of them continued follow-ups of 1, 6, and 12 months. All patients were female with age range of 33-58 years, mean (± SD) age of 44 ± 7.50 years and disease duration of 12.16 ± 4.08 years. VAS score, DAS28ESR, ESR and CRP were measured and analyzed for all 13 patients (Table 1). At months 6 and 12 after MSCs transplantation, VAS score was significantly decreased in comparison with the pre-injection level (P <0.01 and <0.001, respectively) (Figure 1).

**Fig. 2.**
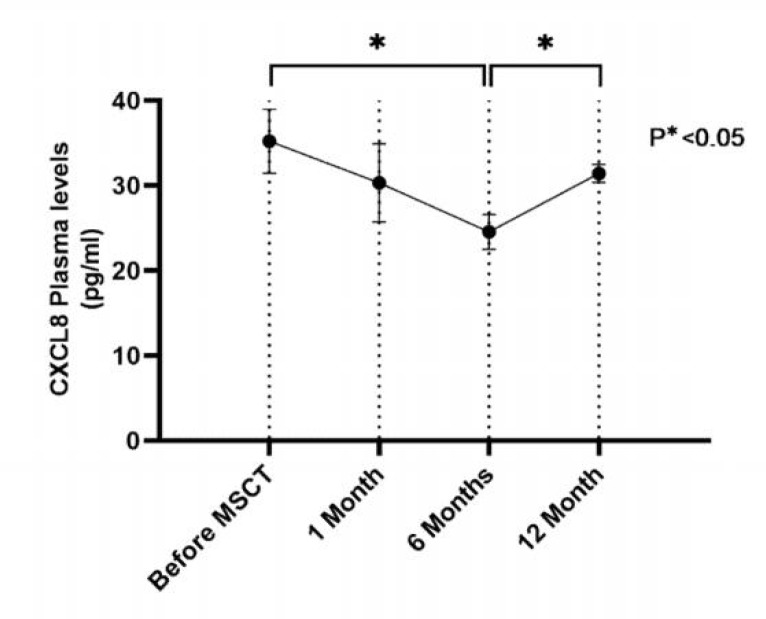
Plasma levels of CXCL8 in patients with RA before and after mesenchymal stem cell transplantation. Data are presented as  mean ± SEM

**Fig. 3:**
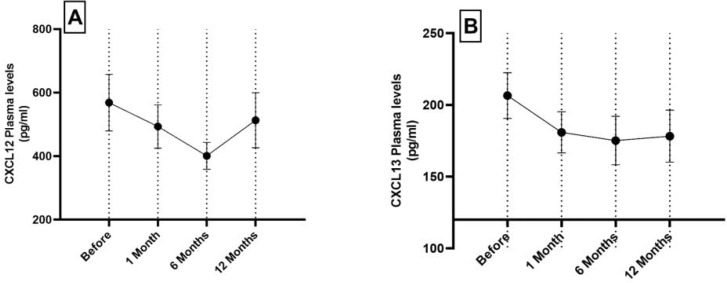
Plasma levels of CXCL12 (A) and CXCL13 (B) in patients with RA before and after mesenchymal stem cell transplantation. Data are presented as mean ± SEM

The plasma CXCL8 levels were decreased during the first month of follow-up in comparison with the pre-injection level, but this decrease was not significant. At month 6 after MSCs transplantation, the level of this chemokine was significantly decreased in comparison with the pre-injection level (P<0.05). The level of this chemokine was significantly increased at month 12 in comparison with month 6 (P<0.05) (Table 2, Figure 2). The plasma levels of CXCL12 and CXCL13 were decreased at follow-ups of 1 and 6 months after MSCs transplantation in comparison with the pre-injection levels, but this decrease was not significant. Moreover, the plasma levels of these chemokines were non- significantly increaesd at month 12 after injection in comparison with months 1 and 6 (Table 2, Figure 3 A and B). There were no significant associations between chemokine levels and clinical and para- clinical examinations.

**Table 1:** Clinical and laboratory characteristics before and after mesenchymal stem cells transplantation

Clinical and laboratory indicators	Before MSCT	1 Month after MSCT	6 Months after MSCT	12 Months after MSCT
**VAS score**	7.92 ± 0.54	6.67 ± 0.56	5.76 ± 0.67^a^ **	5.61 ± 0.70^b^ ***
**DAS28-ESR**	5.56 ± 0.40	5.04 ± 0.44	5.06 ± 0.34	4.72 ± 0.50
**CRP (mg/l)**	14.12 ± 5.09	9.63 ± 3.64	8.53 ± 2.03	9.71 ± 3.64
**ESR (mm)**	23.75 ± 7.73	14.58 ± 4.62	14.58 ± 3.69	15.41 ± 3.74

MSCT: mesenchymal stem cells transplantation; VAS: visual analog scale; DAS28-ESR: disease activity score-28 for RA with erythrocyte sedimentation rate; ESR: erythrocyte sedimentation rate; CRP: C-reactive protein. Data are presented as mean ± SEM. a: ** P<0.01 in comparison with before MSCT; b: *** P <0.001 in comparison with before MSCT.

**Table 2:** Effects of mesenchymal stem cells transplantation on CXCL8, CXCL12 and CXCL13 plasma levels

Plasma Cytokine Concentration	Before MSCT	1 Month after MSCT	6 Months after MSCT	12 Months after MSCT
CXCL8 (pg/ml)	35.21 ± 3.78	30.31 ± 4.59	24.54 ± 2.03^a ^*	31.40 ± 1.07^b ^*
CXCL12 (pg/ml)	568.53 ± 89.04	493.25 ± 68.55	400.83 ± 42.30	513.21 ± 86.82
CXCL13 (pg/ml)	206.51 ± 15.99	180.82 ± 14.40	175.16 ± 16.92	178.23 ± 18.13

MSCT: mesenchymal stem cells transplantation. Data are presented as mean ± SEM. a: * P<0.05 in comparison with before MSCT; b: * P<0.05 in comparison with 6 months after MSCT.

## Discussion

The chemokines are major regulators of cell-cell adhesion and cell trafficking, and are usually involved in cell homing or chemotaxis (22). According to previous studies, the chemokines have an important role in the pathogenesis of RA and there are patterns of myelo-monocyte migration towards inflamed joints in patients with RA (16). The infiltrated leukocytes in the joints and synovial-lining cells are important sources for the production of anti-inflammatory chemokines, such as CCL2, CCL3, CCL5, CCL20, CXCL9 and CXCL10, and also major chemokines for B lymphocyte homeostasis, including CXCL12 and CXCL13 (23, 24). In this study, the plasma levels of CXCL8, CXCL12 and CXCL13 chemokines were investigated before and after injection of autologous MSCs to patients with RA. This study showed that the plasma CXCL8 level was decreased non-significantly during the first month of follow-up in comparison with the pre-injection level.In the month 6 after MSCs transplantation, the level of this chemokine was significantly decreased in comparison with the pre-injection level. However, the level of this chemokine at month 12 was significantly higher than that of month 6. Recent studies have indicated an elevated synovial and serum levels of CXCL8 in patients with RA. The CXCL8, as a neutrophil- recruiting chemokine, is responsible for an increase in the count of neutrophils in the joints of RA, which can cause clinical manifestations of joint swelling and pain. Based on *in vivo* studies, the injection of only one intra-articular unit of CXCL8 could induce the synovial hyperplasia similar to the human RA (16, 25). The neutrophils, as the predominant leukocytes in the arthritis, play a key role in the onset and progression of the RA. Neutrophil-mediated inflammation involves the secretion of pro-inflammatory cytokines, reactive oxygen and nitrogen species, and granules containing degrading enzymes that can further damage tissue and enhance neutrophil response. Thus, the modulation of the neutrophil migration and function is a potential target for the pharmacological interventions related to arthritis (26). A significant decrease in the CXCL8 level at month 6 after injection suggests that after MSCs migrate to the arthritis, these cells may interact with the synovial-lining cells, leading to a decreased production of this chemokine from synovial-lining cells, which can consequently reduce the blood concentration of CXCL8. The decreased production of chemokines by the synovial-lining cells can prevent more immune cells from migrating to the site, and reduce the disease severity. Our results showed a significant increase in the CXCL8 levels at month 12 in comparison with month 6 following injection. In other words, the significant decrease of CXCL8 at month 6 showed an increasing trend throughout the time and almost reached the pre-injection levels at month 12. One of the most possible explanations for this result might be due to the insufficiency in repetition of MSCs injection. It seems that the frequency of injection may be necessary to maintain the immunomodulatory effects of this kind of treatment in suppressing CXCL8 in patients with RA.

Our results indicated that the level of CXCL12 chemokine was decreased non-significantly at months 1 and 6 after MSCs transplantation in comparison with the pre-injection level. In addition, the plasma level of this chemokine was increased at month 12 after injection in comparison with months 1 and 6. The RA synovium plays an important role in the production of inflammatory mediators that enter the bloodstream after production in the synovium. The CXCL12 is a member of the C-X-C subfamily of chemokines that was first isolated from bone marrow stromal cells. RA synovial tissue has been shown to be rich in CXCL12, which is produced by fibroblast-like synoviocytes (27), and this chemokine plays an essential role in B cells resistance to apoptosis, and supports the hypothesis that these synovial cells are involved in the homing and survival of B cells (28). In addition, studies have shown that the plasma cells also migrate toward the CXCL12 in a concentration gradient manner (29). Previous studies have indicated that the synovial and circulating CXCL12 levels are significantly higher in the RA patients than in the healthy subjects (14, 30, 31). It’s possible that MSCs, after migrating to the inflamed areas, affect the CXCL12-producing cells thereby reducing the synovial and circulating production of this chemokine and subsequently reducing the migration of peripheral blood leukocytes to the inflamed area.

On the other hand, the present study demonstrated that the MSCs transplantation non-significantly decreased the CXCL13 level at months 1 and 6 in comparison with the pre-injection level. Furthermore, the plasma level of this chemokine was non-significantly increased at month 12 after injection in comparison with months 1 and 6. Studies on CXCL13 as a chemotactic factor for the B lymphocytes have opened promising windows for finding a valuable prognostic factor in RA. CXCL13 has been implicated in several autoimmune diseases by redistributing B lymphocytes into damaged tissues, reorganizing them into micro anatomic positions and possibly enhancing their activation through  B-cell receptors (10).The serum CXCL13 levels are raised in RA patients in comparison with healthy controls (32, 33). The synovial levels of this chemokine correlate with the serum CXCL13 accumulation in RA patients (34, 35).The CXCL13 appears to be a marker of disease severity in RA, so that a prospective study reported that patients with RA in the early onset of the disease with the highest serum level of CXCL13 had the highest rate of progression of joint damage in long-term follow-up (32, 36). According to the results of our study and regardless of insignificancy between the various time points of the follow-ups, and just by considering the decreasing trend of CXCL13, it is possible that MSCs may react with the CXCL13-producing cells during migration to the inflamed joints and decrease the production of this chemokine. We suggest that this decline may occur initially in the joints and then in the bloodstream of RA patients.

In conclusion, the injection of MSCs may decrease the plasma level of CXCL8 in RA patients at month 6 after transplantation. The interaction of MSCs after migration to the inflamed joints with CXCL8-producing cells could be one of the possible mechanisms that reduce its production in the joints and subsequently in the plasma of RA patients. Also, the decreased production of chemokines could lead to a decrease of the leukocytes migration to inflamed tissues in RA patients. The intravenous injection of MSCs might be an effective therapeutic intervention in the treatment of RA, possibly by modulating the immune system and preventing joint remodeling. As the results show, the effect of MSCs transplantation on CXCL8 level diminished after 12 months and it was returned to the pre-injection level. In order to boost the MSCs transplantation effects, an increase of the dose of MSCs and replication of injections is suggested for further studies.
